# Test–Retest and Between–Device Reliability of *Vmaxpro* IMU at Hip and Ankle for Vertical Jump Measurement

**DOI:** 10.3390/s23042068

**Published:** 2023-02-12

**Authors:** Lamberto Villalon-Gasch, Jose M. Jimenez-Olmedo, Javier Olaya-Cuartero, Basilio Pueo

**Affiliations:** Research Group in Health, Physical Activity, and Sports Technology (Health-Tech), Faculty of Education, University of Alicante, San Vicente del Raspeig, 03690 Alicante, Spain

**Keywords:** intra-session, intersession, between-session, sensibility, countermovement jump, CMJ, agreement, error

## Abstract

The ability to generate force in the lower body can be considered a performance factor in sports. This study aims to analyze the test–retest and between-device reliability related to the location on the body of the inertial measurement unit *Vmaxpro* for the estimation of vertical jump. Eleven highly trained female athletes performed 220 countermovement jumps (CMJ). Data were simultaneously captured by two *Vmaxpro* units located between L4 and L5 vertebrae (hip method) and on top of the tibial malleolus (ankle method). Intrasession reliability was higher for ankle (ICC = 0.96; CCC = 0.93; SEM = 1.0 cm; CV = 4.64%) than hip (ICC = 0.91; CCC = 0.92; SEM = 3.4 cm; CV = 5.13%). In addition, sensitivity was higher for ankle (SWC = 0.28) than for the hip method (SWC = 0.40). The noise of the measurement (SEM) was higher than the worthwhile change (SWC), indicating lack of ability to detect meaningful changes. The agreement between methods was moderate (*r_s_* = 0.84; ICC = 0.77; CCC = 0.25; SEM = 1.47 cm). Significant differences were detected between methods (−8.5 cm, *p* < 0.05, ES = 2.2). In conclusion, the location of the device affects the measurement by underestimating CMJ on ankle. Despite the acceptable consistency of the instrument, the results of the reliability analysis reveal a significant magnitude of both random and systematic error. As such, the *Vmaxpro* should not be considered a reliable instrument for measuring CMJ.

## 1. Introduction

The ability to generate force in the lower body can be considered a performance factor in sports. One way to assess and monitor this force and power is through the vertical jump (VJ) [1]. The countermovement jump (CMJ) has been used to monitor the fitness of athletes [2,3,4] and fatigue [5,6]. There are many protocols and instruments used to carry out the analysis and monitoring of VJ [7], the most reliable of which are those that employ the double integration of reaction forces using force platforms and marker tracking with motion capture systems (MoCAP) [8]. However, these instruments are expensive, complex to set up and calibrate, and difficult to operate, and their use is therefore restricted to research use. They also have the added problem that data cannot be collected outdoors or on certain sport-specific surfaces such as grass or sand, which is also a problem with jump mats [9].

Accelerometers represent an alternative method that partially addresses the limitations of force platforms and motion capture (MoCAP) systems. Due to their smaller size and cost-effectiveness, accelerometers have seen a significant increase in usage in recent years for the study of human movement [10,11]. Inertial measurement units (IMUs) are electromechanical measurement systems that can combine time-accurate data acquisition with algorithms collected from all its sensors (i.e., accelerometer, gyroscope, and magnetoscope) to track position in three dimensions, without affecting the natural movements of athletes [12]. As a result, accelerometers are highly ecologically valid instruments, as they allow for data collection in a less obtrusive manner.

The validity of the *Vmaxpro* has been examined in the context of loaded jumps and has been determined to be valid in these studies [13,14]. However, no validation studies have been conducted specifically for the measurement of unloaded (CMJ) [13,14]. In terms of reliability, IMUs typically exhibit values that are considered reliable. This trend is supported by studies such as that of Rago et al. [15], which analyzed the reliability of the *Myotest* IMU. They detected CV values of 4.2%, test–retest ICC of 0.97, and SEM values of 0.5 cm, as well as SWC of 0.8, indicating this IMU to be a valid instrument. Lower reliability indices were reported by Brooks et al. [16], who detected standardized SEM values of 0.3% which they considered moderate, and test–retest ICC of 0.86. However, the authors still considered the IMU to be reliable, despite the standardized SEM exceeding 0.2%.

Many studies place the IMU on the hip during data collection; however, the findings of Spangler et al. [17] suggest that placement of the IMU on the torso does not significantly affect test–retest reliability, as they observed ICC values of 0.85 and CV of 6.7% for the *Catapult GPS* IMU. In contrast, Rantalainen et al. [18] detected lower reliability values (ICC = 0.686 and CV = 8.7%) for the same device on the torso. Similarly, Garnacho-Castaño et al. [19] placed the *Stride* IMU on the ankle and detected values similar to those on the hip (ICC= 0.90; CV = 4.7%).

The surface on which the jumps are performed can also impact the reliability of the measurements, as surfaces such as sand or grass can introduce additional variability and potentially alter the technical execution of the movement [9]. For example, a study by Schleitzer et al. [20] examined the jumping performance of beach volleyball players on a sand surface using the *Movesense* IMU, and detected ICC values of 0.866 [20]. Hence, studies in which the IMU is not placed on the hip, or those conducted on surfaces that are less stable, tend to have lower reliability values compared to studies where the IMU is placed on the hip. The ICC values are usually above 0.95 and CV values are around 3.5% in these studies [15,21,22,23]. Consequently, IMUs can be considered reliable instruments in the measurement of VJ, although factors such as biological variability, surface, or location of the instrument may influence their reliability [24].

To the best of our knowledge, the reliability of the *Vmaxpro* for measuring VJ has not been evaluated. Additionally, there are currently no studies that have examined the impact of the location of the accelerometer on different body segments for measuring CMJ on reliability. Further research is necessary to determine the reliability of *Vmaxpro* in measuring VJ and to establish whether the instrument can consistently produce measurements that are of sufficient quality to be useful for practitioners. Therefore, the aim of this study is to evaluate the test–retest and inter-device reliability of the *Vmaxpro*, when placed at the ankle or hip, in measuring countermovement jump (CMJ) in highly trained female athletes.

## 2. Materials and Methods

### 2.1. Study Design

This observational study utilized a repeated measures design to determine the test–retest and between-device reliability of the IMU-based *Vmaxpro* for VJ measurements. Data were simultaneously collected by two identical specimens of *Vmaxpro*, located on the right ankle, 1 cm above the malleolus of the tibia, and on the back above the hip between the first and fifth lumbar vertebrae. This design allows to compare the results of the jump estimation obtained by both instruments and to study the absolute error, the degree of agreement and the existence of differences between them. The sample size was determined using G*Power (v3.1.9.7, Heinrich-Heine-Universität Düsseldorf, Düsseldorf, Germany), estimating a minimum of 220 jumps for the Wilcoxon test (α = 0.05, two-tailed, ES > 0.25), and for correlations of two paired variables (power of 90%, α = 0.05, two-tailed and ES > 0.4). For this purpose, 11 participants performed 10 jumps with countermovement, resting 2 min between attempts in each session, resulting in a total of 220 valid jumps.

### 2.2. Procedure

The experimental procedure was conducted in accordance with a randomized, within-subjects design, in which participants were assessed in three separate sessions separated by a seven-day interval. To control for potential effects associated with circadian rhythms, all testing sessions were conducted at the same time of day. During the initial testing session, participants were provided with familiarization of the experimental protocols and anthropometric measurements were collected. In the second and third testing sessions, the same procedures were repeated in the same order: first, a standardized warm-up consisting of five minutes of continuous running was completed, followed by three minutes of dynamic range-of-motion exercises, and then two minutes of familiarization jumps in which subjects were instructed in the initial and final positions of the jump. After the warm-up, a four-minute rest period was implemented during which the inertial device was set up and the jumping protocols were reviewed. Participants then completed 10 CMJ with two minutes of rest between each attempt to control for the effects of fatigue [25].

To avoid displacements in the transverse and frontal plane, take-off and landing jumps were executed completely within the limits. CMJ were executed according to established protocols, with a rapid descent to a depth self-selected by each participant [26,27], followed by an immediate and powerful ascent to achieve take-off [26,27]. All tests were performed with the hands placed on the iliac crests in the Akimbo position [28] to avoid variability generated by the action of the arms. Participants were instructed to jump as high as possible on each attempt, and, in addition, to land on tiptoe imitating the position adopted by the ankle joint in the take-off phase, thus attempting to minimize the error produced by variations in the angle of the ankle flexion in the landing phase [29]. The jumps were monitored by a trained instructor to ensure proper execution, and attempts were deemed invalid if any of particular criteria were not met, namely if the subjects did not land within the established boundaries, if they did not land on the balls of their feet, or if they separated their hands from the iliac crest at any point during the jump. All records were collected simultaneously by two units of the *Vmaxpro*.

### 2.3. Participants

Eleven highly trained female volleyball players [30] from the Spanish Superliga 2 voluntarily participated in this validation study. All participants met the following criteria to be included as highly trained athletes [30]: (i) competing at the national level, (ii) being part of a team competing in the second division of the Spanish national volleyball league (Superliga 2), (iii) completing structured and periodized training and developing towards (within 20%) of maximal or nearly maximal norms within volleyball, (iv) developing proficiency in skills required to perform volleyball. The descriptive data of the sample can be seen in Table 1.

All participants signed an informed consent document informing them of the characteristics of the intervention, as well as the strictly scientific use of the data obtained in the intervention as specified in the World Medical Association (WMA) Declaration of Helsinki; Ethical Principles for Medical Research Involving Human Subjects 1975 (revised in Fortaleza, Brazil in 2013). In addition, this research was approved by the ethics committee of the University of Alicante (UA-2018-11-17).

All participants met the three inclusion criteria for participation in this study: being female, aged over 18 years, having 3 years minimum of training experience in volleyball, being familiar with CMJ. The exclusion criteria included presenting a current or previous pathology that entailed a medical contraindication for physical activity, presenting a previous musculoskeletal injury or one acquired during the experimental phase, not participating in all the interventions included in the study, and ingesting alcohol or drugs in the 48 h before the tests.

### 2.4. IMU-Based Vmaxpro

**Figure 1:**
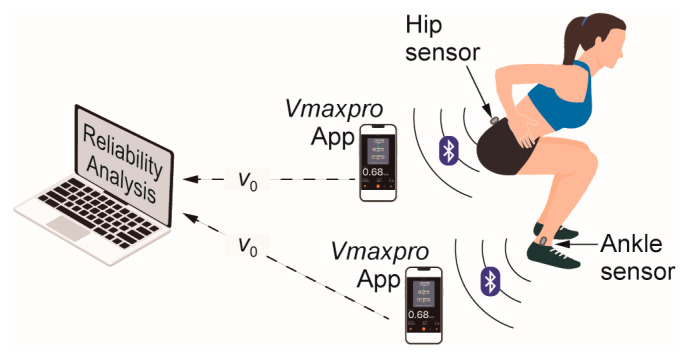
Experimental setup showing the locations of the two *Vmaxpro* devices: hip (close to the center of mass, as indicated by the manufacturer), and ankle (tibial malleolus). Data are sent to smartphones via Bluetooth and the take-off velocity *v*_0_ is used to conduct the reliability analysis.

The *Vmaxpro* (Blaumann & Meyer-Sports Technology UG, Magdeburg, Germany) consists of a triaxial accelerometer, a gyroscope, and a magnetometer, weighing 16 g and measuring 4.5 × 2.7 × 1.2 cm. It has a sampling rate of 1000 Hz [31] and can be attached to metal surfaces by magnets or placed elsewhere using an elastic strap. This IMU is primarily designed for velocity-based resistance training [32], obtaining data from acceleration integration, so it can provide values for related variables such as peak velocity, average velocity, peak eccentric velocity, average eccentric velocity, percentage of force development, percentage of eccentric force development, average propulsive velocity, distance, and duration. The height of a jump can be calculated based on the velocity of the jumper’s center of mass at take-off [33]. By applying the law of conservation of mechanical energy to the flight phase of the jump, a relationship between jump height and take-off velocity can be established. In the case of vertical jumping, air resistance is considered minimal, so the jumper can be treated as a projectile in free flight. Taking into account the changes in kinetic energy and gravitational potential energy from the moment of take-off to the peak of the jump, the jump height reached can be calculated by

$$VJ=\frac{v_{0}^{2}}{2g},$$

where *v*_0_ is the take-off velocity. Therefore, by measuring the peak velocity data with the IMU, the vertical jump height can be calculated as this corresponds to the take-off velocity [1]. The data are sent instantly via Bluetooth wireless connection (65 Hz) to a smartphone or tablet device with the *Vmaxpro* app (BM Sports Technology GmbH, Magdeburg, Germany) installed, allowing the data to be viewed instantly and exported to a spreadsheet in CSV format. Before each measurement, each device was calibrated on all six faces by placing it on a completely flat surface for a sufficient period of time, allowing the software to recognize and establish the local three-dimensional coordinates. Once calibrated, the first unit was placed on an elastic band to be as close as possible to the center of mass, at the subject’s hip, according to the manufacturer’s specifications [34] (hip device). The second unit was placed by securing it with pre-bandage tape on top of the tibial malleolus (ankle device). In this study, a smartphone with the same version of the app was utilized for each sensor to simultaneously collect take-off velocity data on each jump. The paired data were then organized into a spreadsheet format and analyzed using a statistical software package. The complete setup is shown in Figure 1.

### 2.5. Statistical Analysis

Descriptive data are shown as the mean and standard deviation. The Kolmogorov–Smirnov test was used to analyze the normality of the sample, resulting in a non-normal distribution for the *Vmaxpro* data group located at the hip. The reliability of *Vmaxpro* was determined through various tests aimed at estimating the level of agreement and the magnitude of the error in the measure under test–retest and between the different device locations (hip or ankle devices) [35]. Given the non-parametric nature of the sample, the correlation analysis was carried out using the Spearman’s coefficients (*r_s_*),

$$r_{s}=\frac{\mathrm{cov}\left(R\left(X\right),\;R\left(Y\right)\right)}{\sigma_{R\left(X\right)}\sigma_{R\left(Y\right)}},$$

where cov(R(*X*), R(*Y*)) is the covariance and *σ*_R(*X*)_ and *σ*_R(*Y*)_ are the standard deviations of the rank variables. The intraclass correlation coefficient (ICC) (3,1) was used to determine the intra-session reliability of each of the instruments (consistency),

$$\mathrm{ICC}\left(3,1\right)=\frac{MS_{R}-MS_{E}}{MS_{R}+\left(k-1\right)MS_{E}},$$

while ICC (2,*k*) was used to establish the reliability between instruments [36],

$$\mathrm{ICC}\left(2,k\right)=\frac{MS_{R}-MS_{E}}{MS_{R}+\frac{MS_{C}-MS_{E}}{n}},$$

where *MS_R_* and *MS_C_* are the mean square for data in rows and columns, respectively; *MS_E_* is mean square for error; *n* is the number of subjects; and *k* is the number of measurements [36]. The Lin concordance index (CCC) was calculated to determine the degree of agreement between the two device locations,

$$\mathrm{CCC}=\frac{2\rho \sigma_{x}\sigma_{y}}{{\left(\mu_{x}-\mu_{y}\right)}^{2}+\sigma_{x}^{2}+\sigma_{y}^{2}},$$

where *ρ* is the correlation coefficient, *µ* and *σ*^2^ are the means and variances for *x* and *y*. CCC can be split into two terms CCC = *ρ* × *C_b_*, which indicates the degree of similarity between the jump height data obtained from the two devices, where *ρ* represents the precision of the measurement and *C_b_* represents the accuracy of the measurement. An ideal scenario, where *x = y*, would result in a CCC of 1.0 [37]. The results obtained for the different correlation coefficients were classified as trivial (<0.1), small (0.1–0.29), moderate (0.3–0.49), high (0.5–0.69), very high (0.7–0.89), and practically perfect (>0.9) [38].

Additionally, a linear dependence analysis was performed on the paired observations using the Passing–Bablok linear regression method [39]. This method was used to determine the slope and intercept necessary for obtaining the fitting equation between the two instruments. The standard error of the estimate (SEE) was also determined to evaluate the degree of fit of the data to a linear model,

$$\mathrm{SEE}=\sqrt{\frac{\sum {\left(x-x\prime \right)}^{2}}{n}},$$

where *x* is the measured values, *x′* is the values predicted by the multiple regression model and *n* is the number of pairs of measures. The magnitude of the error was estimated by calculating the standard error of the measure SEM,

$$\mathrm{SEM}=\frac{Sd_{diff}}{\sqrt{2}},$$

where *Sd_diff_* is the standard deviation of the difference [40]. This statistic provides information on the error in absolute terms from the analysis of the dispersion of values around the true value [41]. SEM can also be expressed in its standardized form interpreting those values of SEM as trivial (<0.2), small (0.2–0.59), moderate (0.6–1.19), large (1.2–1.99), and very large (>0.2) [41]. The relative reliability of the measurement was established by calculating the coefficient of variation (CV) as

$$\mathrm{CV}=100\cdot \frac{SEM}{\mu },$$

where *µ* is the mean value. The CV outcomes were then classified according to previous studies [38,42] as follows: low (>10%), moderate (5–10%), good (<5%). To determine if the method is highly reliable, it was established that ICC should be greater than 0.90 and CV should be less than 5% [37,41,43].

Sensitivity of the measurement was evaluated using the smallest worthwhile change (SWC), which allows for determining the minimum improvements that present a practical impact [44],

$$\mathrm{SWC}=0.2\cdot \sqrt{2}\cdot \mathrm{SEM},$$

by knowing the SWC, the signal-to-noise ratio can be determined. If the signal-to-noise ratio (SWC/SEM) is greater than unity, the data can be considered reliable [43,44,45].

To determine the significant differences (systematic bias) in the values shown by the two *Vmaxpro* devices placed on hip and knee, a Wilcoxon test for paired samples was performed, along with the bias-corrected Hedges effect size *g* (ES) [46]

$$g=\frac{\mu_{1}-\mu_{2}}{\sqrt{\frac{\left(n_{1}-1\right)s_{1}^{2}+\left(n_{2}-1\right)s_{2}^{2}}{\left(n_{1}-1\right)+\left(n_{2}-1\right)}}},$$

where *µ* and *s* denote the mean and standard deviation of paired samples 1 and 2. This test was used to evaluate the statistical significance and magnitude of the difference between the two devices [46]. The level of significance was established at *p* < 0.05, and the differences expressed as ES were interpreted according to Hopkins et al. [46] as trivial (<0.2), small (0.2–0.59), moderate (0.6–1.19), large (1.2–1.99), very large (0.2–3.99), and huge (>4.0). The degree of agreement between the height data obtained from the two paired devices was evaluated using Bland–Altman plots. These plots allow visualizing the systematic error and the limits of agreement (LoA) for 95%,

$$\mathrm{LoA}=\pm 1.96\cdot SD_{diff}.$$

The maximum allowed differences were calculated from the CV of each method using the following expression √(CV^2^_method1_ + CV^2^_method2_) [47]. The presence of disagreement between the two methods was determined by analyzing the 95% confidence limits of the upper and lower LoA. If the upper limit is below the minimum allowed difference and the lower limit is above the maximum allowed difference, the methods are considered in agreement [48]. Additionally, the presence of proportional error was identified if the Pearson product–moment correlation coefficient (*r*^2^) is greater than 0.1 [41,49]. This information was used to evaluate the level of agreement and identify any potential sources of error between the two devices.

The level of agreement and potential errors between the two devices were calculated in multiple situations. The reliability between the devices was calculated using paired data from devices located at the hip and ankle, and the intra-session reliability of each device was studied using data from each jump and for each device separately. Additionally, the test–retest reliability between sessions was calculated using data from separate sessions separated by seven days.

Statistical analysis was carried out using the *MedCalc* Statistical Software (v 20.100, MedCalc Software Ltd., Ostend, Belgium) and the validity and reliability analysis spreadsheet available in Sportsciences [50].

## 3. Results

Table 2 shows the descriptive results of jump heights from both sessions and locations, expressed as mean and 95% confidence intervals. In addition, the differences between sessions and between devices are shown. Statistically significant differences were observed for the values between devices (hip and ankle) with large ES, while no significant differences were observed between sessions with trivial ES.

### 3.1. Intra-Session Test–Retest Reliability

Intra-session test–retest reliability was calculated by using data from the first five jumps obtained in the first session. The results were obtained by pairing consecutive jumps and determining the mean test score for both devices, i.e., with the IMU at the hip and at the ankle. These results are presented in Table 3.

The ICC values indicated near-perfect test–retest correlations in both cases, higher for the ankle (ICC 0.91 and 0.96 for the hip and ankle, respectively). The CCC values indicated greater reliability for the IMU placed at the ankle (CCC = 0.93) compared to the IMU placed at the hip. Both devices displayed near-perfect precision and accuracy as determined by CCC.

The random error or noise of the measure, quantified by SEM, was determined to be 1.41 cm for hip and 1.00 cm for ankle. In both cases, the standardized SEM was greater than 0.2, indicating that the random error was not insignificant. The relative reliability was determined to be consistent for both instruments, with CV values above 5% observed for both devices (5.10% and 5.13%). The sensitivity of the instruments was determined by the SWC, which was 0.40 cm and 0.28 cm for the hip and ankle, respectively. In both cases, the noise was greater than the SWC, resulting in a signal-to-noise ratio of less than 1.

**Figure 2:**
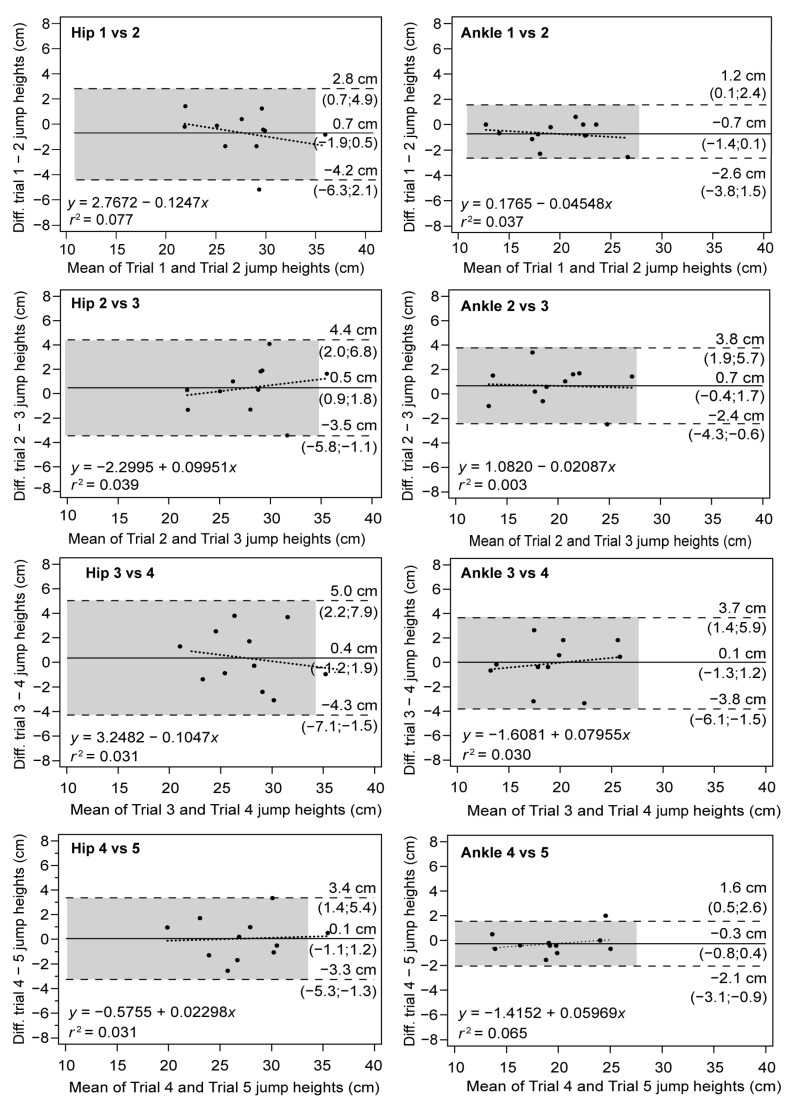
Bland–Altman plots for differences between jumps with the Vmaxpro placed on the hip (**left**) and ankle (**right**). Solid lines: mean differences (systematic error); dashed lines: upper and lower LoA (random error); dotted lines: regression line of the differences between devices.

Consistency between jumps was also studied using Bland–Altman plots, as shown in Figure 2. A high degree of concordance was observed in the test–retest analysis performed on the same day, as nearly all pairings were within the bounds of the upper and lower LoA. Additionally, the systematic error, represented by the mean difference, was determined to be low across all charts, with slightly higher values for the hip (−0.7 to 0.3 cm) than for the ankle (−0.2 to 0.5 cm). No significant trends were observed that suggest heteroscedasticity for a particular device or jump pairing, as the slope values, which indicate proportionality of error, ranged from 5 × 10^−5^ to −0.2. The Bland–Altman plots also reveal wider confidence intervals for the hip, suggesting greater measurement noise in this device.

### 3.2. Between-Session Test–Retest Reliability

The reliability of *Vmaxpro* was assessed through analysis of results obtained in two sessions with a one-week interval. No significant differences were detected between the two sessions in any of the devices, as indicated by the results of the Wilcoxon test (*p* > 0.05; trivial ES). The SEM values for the hip and ankle were 1.5 cm and 1.7 cm, respectively, with trivial (0.1) and moderate (0.46) SEM_std_. The between-session correlations were almost perfect for the hip (ICC = 0.98) and high for the ankle (ICC = 0.79).

### 3.3. Between-Device Reliability (Vmaxpro in Hip vs. Vmaxpro in Ankle)

Table 4 displays the results of the reliability analysis between the devices placed on the hip and ankle. The differences were significant, with a value of −8.5 cm and large ES. The random error was characterized by SEM of 1.47 cm, and a standardized value of 0.4, indicating moderate disagreement (greater than 0.2, the threshold for trivial disagreement). In contrast, the relative reliability was high, with CV values ranging from 14.5% to 19.2% for both groups, and the sensitivity was reflected in the SWC value of 0.4 cm, which represents the minimum jump increment that *Vmaxpro* can detect above the noise of measure.

**Figure 3:**
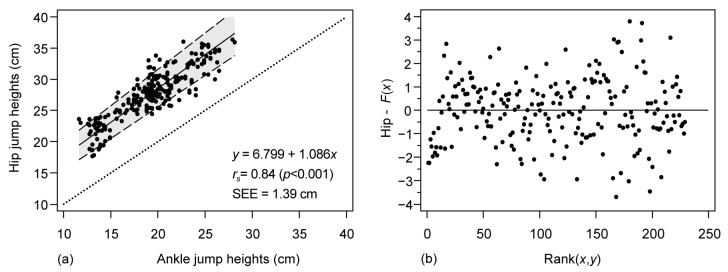
Correlation analysis between the *Vmaxpro* devices placed on hip and ankle through Passing and Bablok regression and residual plot. (**a**) Regression analysis. Solid line: fitted line; dashed lines: 95% CI of the fitted line; dotted line: perfect agreement line, *x* = *y*; *r_s_*: Spearman’s correlation coefficient; SEE: standard error of the estimate. (**b**) Residuals plot.

**Figure 4:**
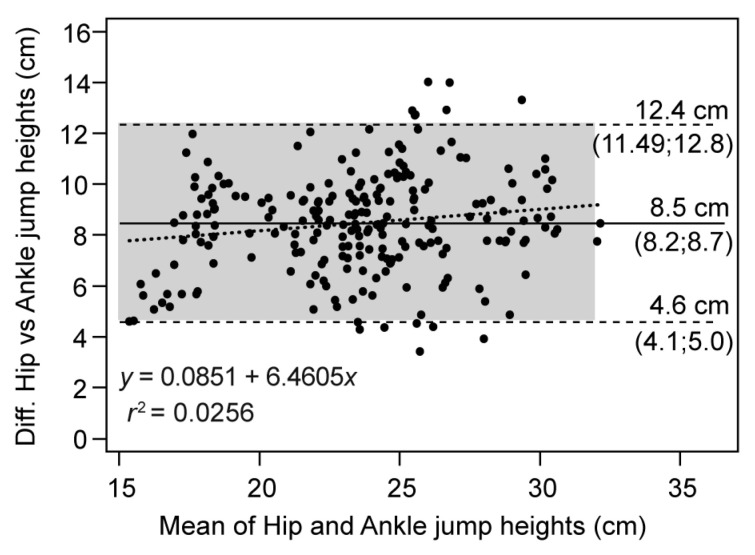
Bland–Altman plot for differences between devices placed on the hip and ankle. Solid lines: mean differences (systematic error); dashed lines: upper and lower LoA (random error); dotted lines: regression line of the differences between devices.

The agreement between the two methods was further analyzed using the Passing and Bablok regression (Figure 3) and the Bland–Altman (Figure 4) plots. The Spearman correlation derived from the regression showed high values (*r_s_* = 0.84, *p*< 0.001), with with *r*^2^ = 0.71. The systematic error between the methods can be quantified using the intercept, which revealed high values of 6.8 cm, a slope of 1.09 (relative error), and random error (SEE) of 1.39 cm. The proportionality of the error was confirmed, as indicated by significant differences in the linearity observed in the Cusum test (*p* = 0.44) and greater dispersion at higher CMJ heights, as visually depicted in Figure 3b.

Additionally, the Bland–Altman plot shown in Figure 4 revealed a high systematic error, with a significant difference (*p* < 0.001) of 8.5 cm (95% CI: 8.20 to 8.72 cm) between devices, and LoA of 4.57 to 12.35 cm. The regression equation showed a slope of 0.0851 (95% CI: 0.02 to 0.15), indicating a degree of proportionality (heteroscedasticity). The differences increased with increasing jump values, but a value of *r*^2^ = 0.03 (less than 0.1) indicated lack of proportionality in the error. Finally, the maximum and minimum difference allowed was ±24 cm with the limits of agreement included in this range, and only 3.4% of the data was outside the limits of agreement.

## 4. Discussion

The aim of the current study was to examine the reliability of the *Vmaxpro* IMU when placed in two different positions, the hip, as recommended by the manufacturer and commonly used in IMU evaluation studies, and the ankle (tibial malleolus), where simultaneous measurements were taken using two devices. The results indicate a variation in the magnitude and consistency of errors between the two placement locations.

The intra-session reliability was assessed through a test–retest design on the same day, analyzing the first five jumps of each device, with the differences between consecutive pairs and the mean of the test being calculated for both hip and ankle devices. Results showed high levels of consistency for the hip with an ICC of 0.91 (ranging from 0.86 to 0.93) and near-perfect reliability for the ankle with an ICC of 0.96 (ranging from 0.92 to 0.98). Additionally, CCC was also high, with values of 0.88 (ranging from 0.831 to 0.92) for the hip and 0.96 (ranging from 0.89 to 0.97) for the ankle. The accuracy values obtained from Lin’s concordance correlation coefficient (*Cb*) were almost perfect in both locations, with a score of 0.99. Precision was determined to be the most significant factor in the final accuracy index, with a score of 0.90 for the hip and 0.95 for the ankle devices. This would mean that the consistency of both devices is affected by the accuracy of the device in making repeated measurements, this phenomenon being more noticeable in the hip placement of the device. The observed consistency values agree with those obtained by Montalvo et al. [21] who determined near-perfect values for the CMJ using the *Push Band* 2.0 IMU with ICC values of 0.98 (95% CI: 0.97 to 0.99). Additionally, the results of other studies indicate higher levels of reliability compared to the *Vmaxpro*. Rago et al. [15] reported ICC values of 0.97 (95% CI: 0.92 to 0.99) for the *Myotest Pro* device in their analysis of six CMJ jumps. The CV values obtained in our study, 5.1% for hip and ankle, are considered moderate and align with the results of other similar studies, where CVs ranged from 4.2% to 7.1% for the CMJ [15,21]. On the other hand, the results of Buchheit et al. [51] showed similar reliability values to the *Vmaxpro* when the device was placed on the tibia, with ICC values of 0.83 and a CV of 5.4%, which are considered reliable.

Regarding the measurement error and sensitivity of the instrument, *Vmaxpro* showed inconsistent values compared to the study by Rago et al. [15], which determined that the device is sensitive enough for vertical jump measurement (SEM = 0.5 cm, SWC = 0.8 cm). However, *Vmaxpro* displayed higher noise values in our study: SEM = 1.4 and 1.0 cm for hip and ankle, respectively, higher than the minimum practically significant value (SWC = 0.4 and 0.3 cm). In contrast, Buchheit et al. [51] determined low standardized SEM values (0.44) and SWC = 3% for ankle device placement, values that align more with *Vmaxpro* (SEMstd = 0.34 to 0.24). The data suggest that small changes can be obscured by the noise and thus affect the instrument’s reliability, so only moderate or large changes can be detected with a single jump. Sensitivity is also much lower than that determined by Rago et al. [15], at 2.8 cm. The standardized SEM values of 0.3 for hip and 0.2 for ankle indicated errors greater than 0.2 and cannot be considered trivial. In general terms, agreement and magnitude values were higher for ankle vs. hip placement (higher ICC, CCC, lower CV, and SEM, lower SWC values).

The Bland–Altman plots confirmed the trends seen in the reliability values. The systematic error low for both the ankle and hip placements, with smaller systematic error observed for the hip (−0.7 to 0.7 cm for the ankle and 0.1 to 0.7 cm for the hip). However, there is a larger dispersion of the data (random error) for the hip, which can be seen in a wider range of agreement in all comparisons to the ankle. Heteroscedasticity was not observed in any method, as all *r*^2^ values are below 0.1. Thus, while the reliability values showed high correlation, the observed noise was still high.

This study highlights that various factors can contribute to measurement noise in IMU assessments. Factors such as the method used to attach the device to the body and variations in detecting the exact take-off moment can affect accuracy and alter the sensitivity of the device [52]. We used an elastic band to attach the IMU at the hip and a tape bandage at the ankle, which may have resulted in more instability at the hip and contributed to the differences in reliability between the two instruments [53]. To improve reliability, it is crucial to control IMUs to minimize fluctuations and avoid disturbing elements that generate noise. Small fluctuations in measurement can lead to significant random error that can compromise reliability.

Reliability of the instrument has been analyzed in a test–retest design in various studies [54]. For *Vmaxpro*, reliability on the hip was determined to be similar (ICC = 0.98; CV = 6.1%) compared to other studies where the device was placed on the hip (ICCs range: 0.86 to 0.98; CVs range: 3.1% to 10.7%) [15,16,19,21,55]. Placing the IMU on the forefoot showed better reliability figures (ICC = 0.89 to 0.90; CV = 4.1% to 4.3%) [18,56] than the *Vmaxpro* on the ankle in this study (ICC = 0.79; CV = 8.1%). The studies that placed the IMU on the torso showed lower reliability values compared to those that placed it on the hip and forefoot (ICC = 0.69 to 0.85; CV = 8.7% to 6.7%) [17,18].

However, the reliability of the instrument should not be solely blamed as the error observed between test sessions may not be entirely due to the instrument. The time period between sessions can cause biological variability that should not be ignored. Such fluctuations in jumping performance can result from changes in physical (fitness, fatigue, learning), psychological (stress, motivation, etc.), and biomechanical (variability in performance technique) factors [54]. These factors can be so significant that they might mask the variability of the instrument. To mitigate this, averaging multiple jumps in the data analysis instead of using raw data can help avoid uncertainty [55].

Once the consistency of the device was evaluated, the agreement between devices was analyzed. A paired jumping study was performed between the two units of the *Vmaxpro* with the device placed at the hip, which is the standardized method, and the ankle. The agreement was considered high with a relative reliability of ICC = 0.77 (95% CI 0.74 to 0.82). However, CCC values were low (CCC = 0.25; 95% CI 0.21 to 0.29) due to lack of accuracy (*C_b_* = 0.29) despite having high accuracy (*ρ* = 0.87). The coefficients of variation were lower for hip (14.5%) compared to ankle (19.2%).

It is observed that studies comparing the reliability of IMU placement during vertical jumping are lacking. However, studies on IMU-collected flight time showed that CVs are lower for placement at the hip (CV < 5.2%) compared to the ankle (11.6%) [15,23,56,57] or torso (CV 6.7% to 7.8%) [17,18]. Placing the device on the forefoot had the lowest CV of 4.7% as observed by Garnacho-Castaño et al. [19], which was lower than the tibia placement above the ankle for *Vmaxpro* but higher than the values observed by Montoro-Bombú et al. of 2.5% [41]. Nevertheless, all placement methods (ankle, hip, torso, and forefoot) were considered reliable [57].

The agreement between devices was assessed through a paired jumping study using two *Vmaxpro* IMU identical units. The paired difference analysis revealed the presence of a high systematic error, leading to a lack of accuracy. The *Vmaxpro* device at the ankle consistently underestimated the hip measurement by approximately 9 cm (*p* < 0.001, ES = 2.2). A previous study by Montoro-Bombú et al. [42] investigated the systematic bias in drop jumps and determined a smaller underestimation of 4.5 cm. The random error (SEM) was determined to be 1.5 cm with a standardized value of 0.4, above the trivial level (0.2), resulting in a sensitivity of 4.1 cm and SWC of 0.4 cm. The Passing–Bablok regression analysis confirmed the trends observed, with a high correlation (*r_s_* = 0.84) and linearity between the two methods. The systematic error was estimated to be 6.7 cm and the slope was 1.09, while the random error was quantified as 1.4 cm using the standard error of the estimate. Although a correlation between the two instruments was indicated, the presence of large systematic and random errors was noted. The Bland–Altman graph showed a systematic error of 8.5 cm, with limits of agreement of 4.6 cm for the lower and 12.4 cm for the upper limit. This implies an underestimation by the ankle instrument and a high degree of dispersion. A proportional error (slope = 0.08) was also observed, but considered trivial (*r*^2^ < 0.1).

The findings of the study indicate a strong linear dependence between the ankle and hip methods, as evidenced by the high correlation results. However, the level of systematic error and noise detected may raise concerns about the reliability of the *Vmaxpro* device in both locations. To the best of our knowledge, there are no studies that specifically examine the reliability of ankle–hip positioning for IMUs. Previous studies on the validity of accelerometer positions on the body have concluded that both ankle and hip positions are acceptable [58]. However, these studies focused on variables related to range of motion rather than dynamic variables like CMJ. In contrast, Althouse [59] conducted a study to determine which body segments or combinations of segments yield the most accurate data for CMJ estimation. Results showed that the root-mean-square error increased as the IMUs were positioned further away from the hypothetical center of mass, with the largest errors observed for accelerations measured in the feet and tibias (15.1 m/s^2^ and 9.0 m/s^2^) compared to those located in the hip or trunk (3.0 m/s^2^). These results differ from those obtained for the *Vmaxpro*, where the magnitude of error was greater for the device located at the hip.

In the design of a reliability study, it is important to recognize that error can stem from two sources: biological variation among subjects and technological variation among items [43]. The objective of these studies is often to compare technological variation; therefore, it is desirable to minimize biological variation. One approach to minimize biological variation is to utilize athletes as subjects, as they tend to exhibit higher reliability compared to non-athletes, regardless of gender. Our study specifically utilized female athletes as subjects, as they meet the criteria of being athletes, and therefore, in our view, testing male participants was not deemed necessary. However, it is recommended that future studies also include male athletes to provide further confirmation of our findings. This would provide a more comprehensive understanding of the relationship between athletic status and reliability in the context of a reliability study.

## 5. Conclusions

The results of this study indicate that the consistency of the *Vmaxpro* shows acceptable values, but the magnitude of systematic and random error observed in the test–retest reliability analysis in the measuring of vertical jump in highly trained female athletes is significant. The location of the device on various body segments impacts the accuracy of the measurement, leading to statistically significant differences when the IMU is placed on the ankle compared to its standard position at the hip. Thus, the inter-device reliability is affected by the placement of the instrument on the body, resulting in an underestimation of the measurement when placed on the ankle.

## Figures and Tables

**Table 1:** Characteristics of highly trained female volleyball players. Data are presented as M ± SD.

N = 11	Mean	SD
Age (years)	23.10	3.10
Height (m)	1.73	0.05
Body mass (kg)	64.0	7.80
Fat percentage (%)	17.30	2.70
BMI (kg/m^2^)	21.30	1.90
Training experience (years)	9.30	1.80

BMI: Body Mass Index; SD: Standard Deviation.

**Table 2:** Descriptive results and differences observed between sessions and between devices (hip and ankle).

Device	Total (cm)	Session 1 (cm)	Session 2 (cm)	Mean Diff. between Sessions (cm)	ES (*g*)
Vmaxpro Hip	27.9	27.1	27.7	−0.1	0.04 (Trivial)
(CI 95%)	(27.1 to 28.65)	(27.2 to 28.6)	(26.9 to 28.5)	(−0.7 to 0.3)	(−0.22 to 0.31)
Vmaxpro Ankle	19.3	19.5	19.4	−0.39	0.07 (Trivial)
(CI 95%)	(18.8 to 19.8)	(18.8 to 20.2)	(18.8 to 19.9)	(−0.9 to 0.1)	(−0.19 to 0.34)
Mean diff. between devices (cm)	−8.5 *	−8.4 *	−8.5 *	–	–
(CI 95%)	(−8.7 to −8.2)	(−8.8 to −7.9)	(−8.8 to −8.1)	–	–
ES (*g*)	−2.2 (Large)	−2.2 (Large)	−2.2 (Large)	–	–
(CI 95%)	(−2.5 to −1.8)	(−2.5 to −1.9)	−2.5 to −1.9)	–	–

* Significant difference for 95% confidence interval (*p* < 0.001); CI = confidence interval; ES = Hedge’s effect size.

**Table 3:** Intra-session test–retest reliability (intra-session consistency) for hip and ankle devices.

	Hip					Ankle				
	2–1	3–2	4–3	5–4	Mean	2–1	3–2	4–3	5–4	Mean
Mean change (cm)	0.70	−0.48	−0.37	−0.05	–	0.71	−0.67	0.07	0.25	–
CI-95% lower	−0.51	−1.83	−1.97	−1.19	–	0.05	−1.74	−1.21	−0.37	–
CI-95% upper	1.91	0.87	1.23	1.09	–	1.38	0.39	1.35	0.87	–
ICC	0.92	0.90	0.86	0.93	0.91	0.98	0.95	0.92	0.96	0.96
CI-95% lower	0.72	0.65	0.53	0.75	0.80	0.92	0.82	0.73	0.89	0.89
CI-95% upper	0.98	0.97	0.96	0.98	0.97	0.99	0.99	0.98	0.99	0.99
CCC	0.89	0.88	0.83	0.92	0.88	0.96	0.92	0.89	0.97	0.93
CI-95% lower	0.66	0.62	0.51	0.92	0.68	0.86	0.74	0.67	0.90	0.79
CI-95% upper	0.96	0.99	0.99	0.99	0.98	0.98	0.93	0.99	0.99	0.97
*ρ* (precision)	0.91	0.89	0.83	0.84	0.90	0.97	0.93	0.91	0.97	0.95
*C_b_* (accuracy)	0.98	0.99	0.97	0.99	0.99	0.98	0.99	0.98	0.99	0.98
SEM (cm)	0.70	1.12	1.35	0.65	1.41	1.19	1.29	1.08	1.15	1.00
CI-95% lower	0.49	0.78	0.94	0.46	1.13	0.90	0.97	0.82	0.87	0.80
CI-95% upper	1.22	1.97	2.37	1.14	1.88	1.80	1.95	1.64	1.74	1.33
SEM_std_	0.32	0.36	0.44	0.29	0.34	0.17	0.27	0.34	0.17	0.24
CI-95% lower	0.23	0.25	0.31	0.20	0.27	0.12	0.19	0.24	0.12	0.19
CI-95% upper	0.57	0.63	0.77	0.51	0.45	0.30	0.47	0.59	0.29	0.32
CV (%)	4.64	5.04	6.08	4.64	5.10	3.63	5.62	7.00	3.63	5.13
SWC (cm)	0.36	0.40	0.48	0.34	0.40	0.20	0.32	0.38	0.18	0.28
CI-95% lower	0.25	0.28	0.33	0.24	0.32	0.14	0.22	0.27	0.13	0.23
CI-95% upper	0.63	0.71	0.84	0.60	0.53	0.35	0.56	0.67	0.32	0.38

CI = confidence intervals for 95%; ICC = intraclass correlation coefficient; CCC = Lin’s concordance coefficient; *ρ* = CCC-derived precision; *C_b_* = CCC-derived accuracy; SEM = standard error of measurement; SEM_Std_ =standardized standard error of measurement; CV = coefficient of variation; SWC = smallest worthwhile change.

**Table 4:** Between device reliability for the *Vmaxpro* inertial measurement unit.

	Ankle vs. Hip Devices	95% CI
Paired differences (cm)	−8.50 *	−8.7 to −8.2
ES (paired)	−2.2	−2.5 to −1.8
ICC	0.77	0.74 to 0.82
CCC	0.25	0.21 to 0.29
*ρ* (precision)	0.87	–
*C_b_* (accuracy)	0.29	–
SEM (cm)	1.47	1.33 to 1.66
SEM_std_	0.39	0.35 to 0.44
CV_hip_ (%)	14.5	–
CV_ankle_ (%)	19.2	–
SWC (cm)	0.42	0.37 to 0.47
SNR	0.28	0.26 to 0.30

95% CI = confidence intervals for 95%; ES = effect size; ICC = intraclass correlation coefficient; CCC = Lin’s coefficient of concordance; SEM = standard error of measurement; SEM_Std_ = standardized SEM; *ρ* = precision derived from CCC; *C_b_* = accuracy derived from CCC; CV = coefficient of variation; SWC = smallest worthwhile change. SNR = signal to noise ratio; * Statistically significant differences (*p* < 0.001).

## Data Availability

The data presented in this study are available on reasonable request from the corresponding author.
